# A Static Phantom for Harmonization of Hyperpolarized [1– ^13^C]Pyruvate Metabolic Magnetic Resonance Imaging

**DOI:** 10.1002/mrm.70442

**Published:** 2026-05-24

**Authors:** Kristina P. Jacobsen, Ingeborg S. Skre, Rie B. Olin, Mathilde H. Lerche

**Affiliations:** ^1^ Section for Magnetic Resonance, Department of Health Technology Technical University of Denmark Kgs. Lyngby Denmark

**Keywords:** ^13^C MRI, harmonization, hyperpolarization, multicenter studies, phantom

## Abstract

**Purpose:**

To develop and characterize a static, reusable ^13^C phantom designed to assess and support the harmonization of MRI setups used for hyperpolarized (HP) [1‐^13^C]pyruvate imaging.

**Methods:**

The phantom was designed as a 200‐mm‐diameter polycarbonate sphere filled with 8M natural abundance urea and incorporating distinct modules for assessing field and SNR homogeneity, geometric fidelity, spatial resolution, and metabolite quantification. The structural design, materials and chemical composition were selected to match the current technical requirements and capabilities in HP [1‐^13^C]pyruvate MRI. The phantom was characterized on a 3T clinical MR scanner to demonstrate representative use cases.

**Results:**

The phantom enabled assessment of field homogeneity ($B_{0}$ variation was approximately 20 Hz and $B_{1}^{+}$ relative flip angle was 1.0 ± 0.1) and Signal‐to‐Noise Ratio of the MRI setup. Spectral‐spatial (SPSP) spiral imaging of the *geometric* module and *resolution* module demonstrated how post‐processing pipelines can influence spatial fidelity and effective resolution. The *quantification* module, containing compounds with chemical shifts matching those of pyruvate and its principal metabolites and mixed in physiologically relevant ratios, enabled metabolite ratio quantification using both CSI and SPSP spiral imaging.

**Conclusion:**

Initial characterization demonstrates that the proposed ^13^C phantom is a practical and versatile tool for benchmarking HP ^13^C MRI system performance, thereby supporting harmonization in multicenter clinical HP ^13^C MRI studies.

## 1 Introduction

Hyperpolarized (HP) ^13^C magnetic resonance imaging (MRI) enables real‐time, non‐invasive measurement of metabolic reactions in vivo. Among available HP probes, [1–^13^C]pyruvate is currently the most extensively studied because of its central role in cellular energy pathways and its robust polarization behavior [1, 2]. HP ^13^C MRI has been applied in a growing number of clinical investigations spanning oncology [2, 3, 4, 5], cardiac metabolism [6], and neurodegenerative disorders [7], with more than 1200 participants scanned across 15 international sites to date [8]. Despite this progress, most studies include only small cohorts, typically 5–10 participants [4, 9, 10, 11], limiting statistical power and clinical generalizability. Larger multicenter studies are needed to establish disease‐specific metabolic signatures, assess reproducibility, and support regulatory evaluation [12].

Conducting multicenter HP ^13^C MRI studies introduces sources of substantial technical variability. Differences in polarization hardware, scanner platforms, gradient performance, RF coil configurations, acquisition protocols, and reconstruction pipelines [8, 13] can influence *B*_0_/*B*_1_ homogeneity, flip‐angle accuracy, spatial resolution, spectral separation, and metabolic quantification [14, 15]. If unaccounted for, these sources of variability can obscure biological effects, introduce systematic site‐dependent biases, and complicate data pooling.

Similar multicenter challenges have been encountered as other quantitative imaging modalities approached broader clinical use. In diffusion MRI, systematic scanner‐ and vendor‐dependent biases motivated harmonized protocols and statistical correction approaches such as ComBat [16, 17]. Quantitative PET likewise demonstrated sensitivity to calibration, acquisition timing, and reconstruction methods, prompting standardized phantoms (e.g., NEMA IQ) and coordinated cross‐site quality‐assurance programs [18]. These experiences underscore the importance of structured QA procedures and standardized reference measurements for achieving reproducible quantitative imaging across sites.

With increasing clinical adoption and a growing number of participating centers, HP ^13^C MRI is entering the same phase of methodological maturation with focus on harmonization. In ^1^H MRI, harmonization is supported by widely used phantoms such as the ACR MRI phantom [19], the ADNI phantom [20], and the ISMRM/NIST system phantom [21], which provide well‐characterized reference features for evaluating geometric accuracy, image uniformity, $B_{1}$ homogeneity, relaxation times, proton density/SNR, and spatial resolution. In contrast, HP ^13^C MRI lacks phantoms with comparable features. Existing designs fall broadly into dynamic [22, 23, 24] and static [13, 25, 26, 27, 28] categories. Dynamic phantoms, which model enzymatic conversion of [1–^13^C]pyruvate to [1–^13^C]lactate, offer physiological relevance but require refilling, exhibit limited stability, and depend on conditions such as temperature and pH, making them impractical for routine QA. Static phantoms are more commonly used but are often strict purpose‐built, vary in geometry and ^13^C substrates. A low‐cost and open‐source design for a durable ^13^C phantom has recently been published in an attempt to provide a common reference tool [28]. While this and other static phantoms represent important steps toward standardization, they are not specifically designed to assess field homogeneity, geometric fidelity, and spatial resolution. Vendor provided natural‐abundance ^13^C phantoms offer consistency but provide only partial support for evaluating the performance metrics relevant to multicenter HP ^13^C studies.

To address these gaps, we here present a static phantom designed specifically for HP [1–^13^C]pyruvate MRI. The phantom provides a reproducible, ^13^C‐specific reference for multicenter calibration and quality assurance. It is designed to support (1) assessment of field and SNR homogeneity, (2) characterization of geometric fidelity or distortions, (3) evaluation of actual spatial resolution across sequences and reconstruction pipelines, and (4) testing of metabolite‐ratio quantification performance.

## 2 Methods

### 2.1 Phantom Design

#### 2.1.1 Geometry and Features

**FIGURE 1:**
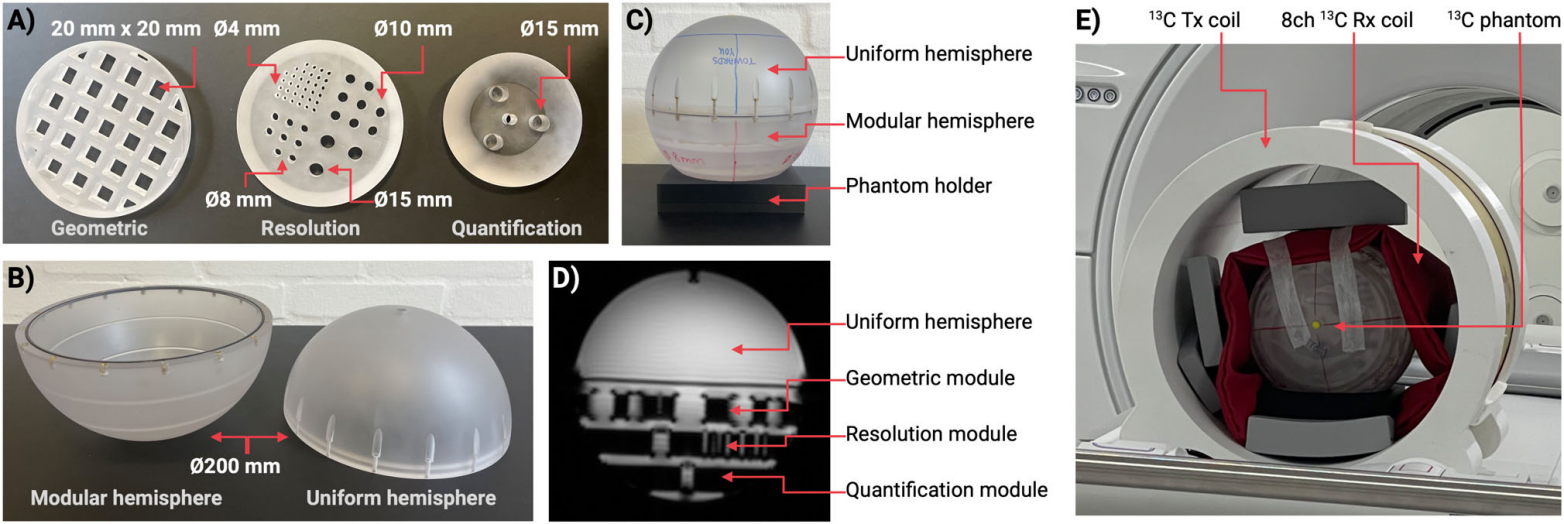
The ^13^C phantom. (A) *Modular* hemisphere modules: a *geometric* module: Grid size 20 mm × 20 mm, wall thickness 10 mm. A *resolution* module: Ø4, Ø8, Ø10, and Ø15 mm holes, each spaced apart by a distance equal to its diameter. A *quantification* module: Three compartments (3.2 mL, Ø15 mm) containing [1–^13^C]glycine‐d_5_, [1–^13^C]propionate, and ^13^C urea in different ratios. (B) Polycarbonate *modular* and *uniform* hemispheres with a solution of 8 M urea. (C) The assembled phantom. (D) Cross‐sectional ^1^H image of the phantom positioned as shown in (C). (E) Phantom placement with ^13^C Rx coil wrapped around and inserted into Tx coil. Foam padding was added between the Rx coil and Tx coil to keep the Rx coil as close to the phantom as possible.

The phantom was designed as a 200mm‐diameter sphere to fit within standard clinical head coil configurations and to provide a uniform magnetic environment during imaging. The structure consists of two hemispheres, Figure 1B, separated by a thin poly(methyl methacrylate) (PMMA) disk. This design allows one hemisphere to be removed or modified without altering the other. The *uniform* hemisphere contains a single homogeneous liquid‐filled cavity, while the *modular* hemisphere houses three 20‐mm disks—*modules*—each incorporating features tailored to specific image‐quality assessments. The modules are separated by 7.25‐mm liquid layers that allows for decoupling, so that each module pattern can be imaged without interference.

Each part of the phantom serves a distinct purpose: The *uniform* hemisphere provides a large, uniform volume for assessing $B_{0}$/$B_{1}$ homogeneity and SNR of the ^13^C coil setup. Of the three disks constituting the modular hemisphere, the *Geometric* module (innermost), Figure 1A, contains a grid pattern (10 mm beams, 20 mm spacing) designed to evaluate geometric differences and distortions under different ^13^C acquisition schemes. The *Resolution* module (middle): includes circular holes with diameters of Ø4 mm (33), Ø8 mm (8), Ø10 mm (8), and Ø15 mm (3), hole spacing is equal to their diameter to minimize partial‐volume overlap. This module enables characterization of achievable spatial resolution, covering a range of relevant in‐plane spatial resolutions in current HP ^13^C MRI. The *Quantification* module (outermost): features three 3.2 mL, Ø15‐mm cylindrical compartments containing ^13^C‐labeled compounds chosen to mimic the chemical‐shift separations of [1–^13^C]pyruvate and two of its metabolic products (lactate and bicarbonate), enabling evaluation of metabolite‐ratio accuracy and dynamic range. An additional through‐hole ensures continuous fluid exchange across the *modular* hemisphere.

#### 2.1.2 Materials and Chemical Contents

The phantom was fabricated from semi‐transparent polycarbonate (PC) (TECANAT, Ensinger) using CNC machining (Gildemeister CTX beta 1250 TC). PC was chosen for its machinability, mechanical stability, and optical transparency, allowing visual inspection of the internal structures and filling process. The separating disk was laser‐cut from PMMA (Plexiglas, Rias). Both hemispheres were filled with an 8 M natural‐abundance urea (90 mM ^13^C) solution prepared in phosphate‐buffered saline (DPBS/Modified, Cytiva) to obtain sufficient loading, conductivity, and isotonicity. Gadolinium (6.9 g/L, Omniscan, GE Healthcare) and sodium azide (0.02% w/v, 99.5%, Sigma‐Aldrich) were added to ensure suitable relaxation times ($T_{1}$ ≈100 and $T_{2}$ ≈55 ms, see Tables S1 and S2 for further details) and prevent microbial growth.

The *quantification* module compartments were filled with mixtures of [1–^13^C]glycine‐d_5_ (99% ^13^C, 98% D, Cambridge Isotope Laboratories), [1–^13^C]propionate (Sodium propionate, 99% ^13^C, Sigma‐Aldrich), and ^13^C urea (99% ^13^C, Cambridge Isotope Laboratories). Three mixture ratios were prepared: 0.5: 0.5: 0.5 M, 1.0: 0.4: 0.3 M, and 2.0: 0.5: 0.2 M chosen to span low, moderate, and high concentration difference regimes relevant for metabolite‐ratio testing. All mixtures were dissolved in PBS doped with gadolinium (8 ± 1 mM, Dotarem, Guerbet) to adjust relaxation times: $T_{1}$ values approximately 300, 800, and 230 ms for [1–^13^C]glycine‐d_5_, [1–^13^C]propionate, and ^13^C urea, respectively and $T_{2}$ values approximately 120, 70, and 60 ms—additional details are provided in Tables S3 and S4. Sodium azide (0.02% w/v, 99.5%, Sigma‐Aldrich) was added prior to sterile filtration (0.20 μm).

#### 2.1.3 Assembly and Stability

The upper hemisphere was bonded to the PMMA disk using adhesive (Permalock ME 935, Hoejstrup Industrilim), filled via a dedicated port, and sealed with a rubber stopper (Ø6.5–9.5 mm). The *quantification* compartments were filled and sealed individually with glued lids. The three modules were fixed into recessed slots in the *modular* hemisphere to ensure alignment and mechanical stability. The two hemispheres were joined using a rubber O‐ring and fastened with 16 brass M3 screws. The *modular* hemisphere was filled through a second port, sealed with a matching stopper, and excess material trimmed.

As the 8 M urea solution could be prone to precipitation below 5°C, the phantom was stored and handled at room temperature. Stability tests with varying concentrations conducted over a period of 6 months are reported in the Supporting Information.

### 2.2 Imaging Tests for Characterization

All imaging was performed on a 3T clinical scanner (Signa Premier, GE Healthcare). ^1^H imaging used the built‐in body coil, while ^13^C imaging used a custom‐built Helmholtz transmit (Tx) coil (Ø360 mm, length 300 mm) and an eight‐channel HyperFlex receive array [29] positioned as close to the phantom as possible, Figure 1E. The phantom was centered in the Tx coil using a custom 3D‐printed holder. The phantom was positioned with the *uniform* hemisphere oriented toward the scanner bore (patient superior direction), such that the modules were aligned in the axial plane for all imaging except of the *quantification* module. For measurements of the *quantification* module, the phantom was reoriented with the *modular* hemisphere facing upward (patient anterior direction), placing the modules in the coronal plane. Structural reference images were acquired using a 2D SPGR sequence. Prior to imaging, automatic linear shimming was performed, and for ^13^C imaging, transmit gain was calibrated using Bloch‐Siegert phase‐shift [30].

Field homogeneity was assessed using ^1^H $B_{0}$ maps of the *uniform* hemisphere and the modules, and ^13^C $B_{1}^{+}$ maps of the *uniform* hemisphere were acquired using a CSI‐based Bloch‐Siegert approach [31] with a scan time of 13 min. A 2D phase‐encoded CSI acquisition was used to map ^13^C SNR with a scan time of 26 min.

All three modules in the *modular* hemisphere were imaged using a 2D spectral‐spatial (SPSP) spiral sequence [32] (15 mm slice thickness), with individual compounds imaged using frequency‐selective excitation. The *quantification* module was additionally acquired using phase‐encoded CSI and slice‐selective excitation without spatial encoding (spectrum). Scan times were 26 min for the *geometric* and *resolution* modules, and 56 min for the *quantification* module.

Reconstruction and analysis were performed in MATLAB (2025b) using MNS Research Pack tools (GE Healthcare). Images were Gaussian‐filtered (10 Hz), coil‐combined using polynomial‐smoothed low‐pass sensitivity maps [33], and interpolated to match the ^1^H reference. In general, seven out of eight coil channels were included in the analysis, as one channel was known to be nonfunctional. The phantom was positioned such that the remaining functional coil elements provided optimal coverage. For the *quantification* module, only three coil channels were utilized due to low signal from the remaining channels. ROIs were segmented from ^1^H images using intensity thresholding. SNR was computed voxel‐wise from the CSI complex data: Signal was defined as the root‐sum‐of‐squares of each spectrum within a fixed frequency window (600 Hz) centered on the urea peak, while the noise was estimated as the standard deviation of signal‐free spectral regions at the edges (15% on each side) of the complex spectrum.

For the *resolution* module, post‐processing included various Gaussian filters and GL‐HOSVD denoising [34, 35].

Metabolite ratios were calculated from SPSP spiral images as mean compartment signal relative to [1–^13^C]glycine‐$d_{5}$. CSI data were analyzed using AMARES‐based fitting [36] as implemented in OXSA [37], with fitted amplitudes used for ratio estimation.

Additional MRI acquisition parameters are provided in the Supporting Information. 3D CAD models of the phantom and holder are available at https://doi.org/10.5281/zenodo.18225650.

## 3 Results

**FIGURE 2:**
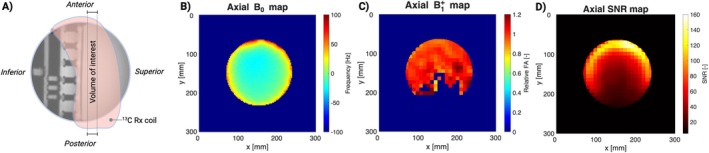
$B_{0}$, $B_{1}^{+}$, and SNR maps of same volume in *uniform* hemisphere of the phantom (the slice is slightly offset from the phantom center, corresponding to a 190 mm cross‐sectional diameter). (A) Schematic of phantom, Rx coil placement, and slice location. (B) $B_{0}$ map at ^1^H frequency range. (C) $B_{1}^{+}$ map shown as relative flip angle. The scan time only provided sufficient SNR to map $B_{1}^{+}$ as shown which visibly correlates with the high‐SNR area of the SNR map and the position of the Rx coil. (D) SNR map (using combination of seven of eight receive coils).

The *uniform* hemisphere enabled assessment of field and SNR homogeneity. The $B_{0}$ variation was approximately 20 Hz (standard deviation at ^1^H frequency), with the largest deviations observed near the phantom's perimeter (Figure 2B). $B_{1}^{+}$ mapping demonstrated field homogeneity (relative flip angle mean = 1.0, standard deviation = 0.1) consistent with the Helmholtz coil geometry (Figure 2C). The SNR distribution followed the expected receive‐coil sensitivity profile (Figure 2D), and was comparable to that obtained from a vendor provided phantom (Figures S2–S5), confirming suitability for receive‐coil characterization.

**FIGURE 3:**
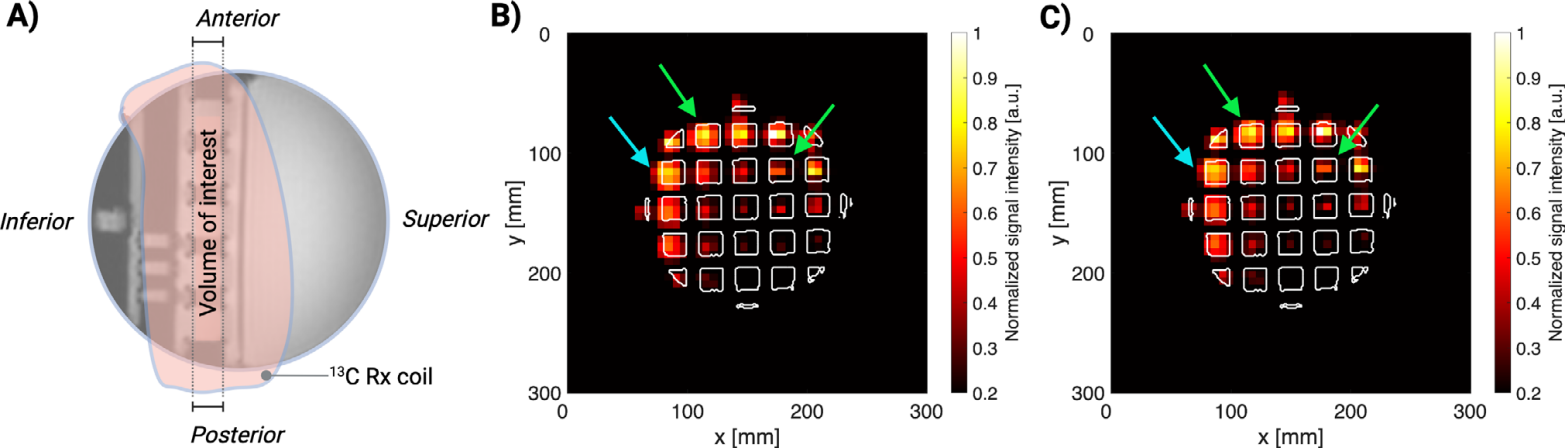
*Geometric* module. (A) Schematic of phantom, Rx coil placement, and slice location. (B) ^13^C image data without half‐voxel shift relative to ^1^H reference (white lines). (C) ^13^C image data with half‐voxel shift. Green arrows highlight example areas of improved consistency from the voxel‐shift; the blue arrow highlights an example of partial volume effects.

The *geometric* module enabled visualization of spatial displacement between the ^13^C signal and the ^1^H reference. Introducing a half voxel shift improved alignment consistency, Figure 3B,C. In both cases, partial‐volume effects were more pronounced in regions of higher SNR, reflecting the receive coil sensitivity profile.

**FIGURE 4:**
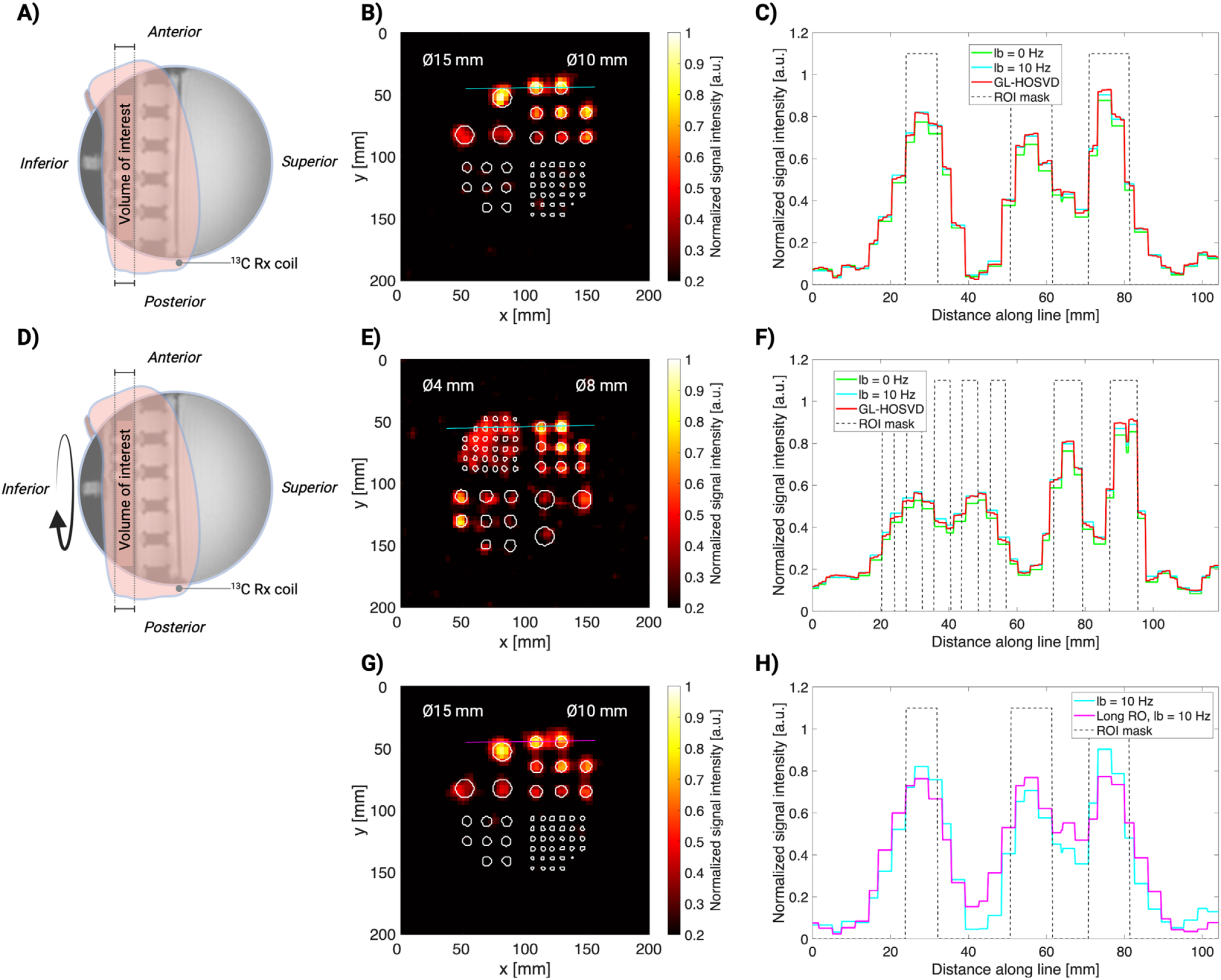
*Resolution* module. (A) Schematic of phantom, Rx coil placement, and slice location for imaging Ø15‐ and Ø10‐mm holes. (B) ^13^C image with 10 Hz line broadening and corresponding ^1^H reference (white). The line profile (blue in ^13^C image) through the Ø15‐ and Ø10‐mm holes is shown in (C). (C) Line profiles with 0 Hz line broadening, 10 Hz line broadening and GL‐HOSVD denoising. Black dashed lines indicate hole locations derived from the ^1^H reference. (D) Schematic of phantom placement and slice location for imaging Ø4‐ and Ø8‐mm holes—the phantom is rotated 180° compared to (A). (E) ^13^C image with 10 Hz line broadening of the Ø4‐ and Ø8‐mm holes. (F) Line profiles of the Ø4‐ and Ø8‐mm holes. (G) ^13^C image from spiral acquisition with longer read‐out duration (45.6 ms) with 10 Hz line broadening (phantom placed as shown in A). (H) Line profile comparisons between the long and the default read‐out (25.5 ms) durations.

In the *resolution* module, the Ø10‐ and Ø15‐mm holes were fully separable for the chosen sequence and reconstruction as indicated by approximately 50% signal reduction between their corresponding peaks (Figure 4C). The Ø8‐mm holes also, exhibited a 50% valley between peaks (Figure 4F), indicating an achievable in‐plane resolution for this sequence and reconstruction close to, or slightly better than, 8 mm, within expectations with a nominal resolution of 6.25 mm. Across reconstructions, GL‐HOSVD denoising yielded slightly higher signal intensities than line broadening approaches while maintaining comparable valley depths (Figure 4C,F). Increasing spiral‐readout duration from 25.5 to 45.6 ms increased signal blurring between holes, Figure 4G,H.

**FIGURE 5:**
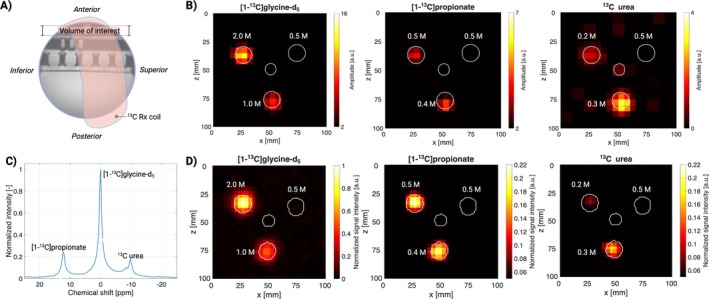
*Quantification* module. (A) Schematic of phantom, Rx coil placement, and slice location. (B) Representative ^13^C CSI images of each metabolite with amplitude values obtained from AMARES‐based fitting. (C) Spectrum of entire *quantification* module. Chemical shift differences between [1–^13^C]glycine‐d_5_, [1–^13^C]propionate, and ^13^C urea are 12 and −10 ppm, respectively. The difference in signal intensity is due to different ratios of metabolites. (D) Representative SPSP spiral images of each metabolite.

A ^13^C spectrum acquired from the *quantification* module demonstrated clear chemical shift separation of all three peaks, with signals from [1–^13^C]propionate located 12 ppm from [1–^13^C]glycine‐d_5_ and ^13^C urea at −10 ppm relative to [1–^13^C]glycine‐d_5_, matching the expected separation of carboxylate carbons in pyruvate and its two metabolites, lactate and bicarbonate (Figure 5C). Using both CSI and SPSP spiral imaging, two of the three compartments were clearly distinguished for each metabolite (Figure 5B,D). Signal intensities of the 0.5: 0.5: 0.5 M compartment were below the SNR threshold due to excessive gadolinium induced line broadening and were therefore excluded from quantification. Residual signal from surrounding hemisphere urea was evident, due to imperfect slice profile selectivity (not visible in figure).

Longitudinal measurements conducted within a four‐month interval confirms good temporal stability of the phantom and ability to obtain robust measurements characterizing the MRI setup. $B_{0}$, $B_{1}^{+}$, and SNR maps all demonstrated consistent results for signal distribution and values (less than 5% difference in $B_{0}$ standard deviation and $B_{1}^{+}$ relative flip angle). For the *quantification* module, the chemical shift between metabolites remained constant, and [1–^13^C]glycine‐d_5_ and [1–^13^C]propionate signal ratios were within 10% of expected concentration ratios—^13^C urea ratio values were influenced by residual signal from the hemisphere and thus sometimes overestimated dependent on exact slice and coil placement.

## 4 Discussion

### 4.1 A Comprehensive ^13^C System Assessment

In this work, we developed and characterized a reusable static phantom specifically designed to support harmonization of HP ^13^C MRI across imaging sites. The phantom integrates four complementary test modules, $B_{0}$/$B_{1}$ homogeneity and SNR assessment, geometric characterization, spatial resolution characterization, and metabolite ratio evaluation, targeting sources of variability known to affect quantitative HP ^13^C measurements.

Because field homogeneity and coil SNR comparisons require identical geometry and chemical composition, a standardized phantom provides a robust basis for inter‐site benchmarking. Our results demonstrate that this phantom captures field and SNR inhomogeneities comparable to those observed with existing vendor provided phantoms within similar scan times. As $B_{0}$/$B_{1}$/SNR mapping and geometric evaluation occur using the same physical object (same geometry, loading, materials, etc.), biases in one domain (e.g., nonuniform excitation) can be further interpreted in the context of geometric or spectral distortions. These phantom properties allow users to make informed adjustments, for example, modifying flip‐angle schemes, selecting more robust shim strategies, revising reconstruction pipelines, and so forth.

The *geometric* module is an “add‐on” compared to existing ^13^C phantoms. It spans a relatively large FOV, enabling evaluation of displacements and distortions across the imaging plane. This makes it well suited for the assessment of inhomogeneity correction procedures. The grid size and wall thickness were chosen to match the coarser spatial resolutions in HP ^13^C images [13].

The *resolution* module enables assessment of achievable spatial resolution under clinically relevant acquisition strategies. The Ø4–Ø15 mm features correspond to reported in‐plane resolutions in recent clinical HP ^13^C studies (brain: 6 × 6 to 20 × 20 ${\mathrm{mm}}^{2}$ [38, 39, 40]; abdomen: 8 × 8 to 22 × 22 ${\mathrm{mm}}^{2}$ [41, 42, 43, 44]) providing a practical benchmark aligned with current and anticipated clinical protocols. However, the urea signal is not intended to replicate metabolite specific $T_{2}^{*}$ behavior and therefore does not report the achievable in vivo resolution, which depends on metabolite‐specific relaxation, signal dynamics, and SNR.

Finally, the *quantification* module enables evaluation of metabolite ratio accuracy. The measured signal ratios reflects the actual concentration ratios (2.0: 0.5: 0.2 M and 1.0: 0.4: 0.3 M for [1–^13^C]glycine‐d_5_, [1–^13^C]propionate and ^13^C urea, respectively), Figure 5B,D. The module also enables evaluation of spectral selectivity, and off‐resonance behavior, all central to pyruvate imaging. The $T_{2}$ (and $T_{2}^{*}$) values of the phantom compounds ([1–^13^C]glycine‐d_5_, [1–^13^C]propionate, ^13^C urea) were intentionally formulated within a broadly relevant range ($T_{2}$ between ∼60 and 120 ms, Supporting Information) rather than to match specific physiological values. In vivo, $T_{2}^{*}$ values vary substantially by metabolite, tissue environment, and timing, with reported ranges of approximately 20–75 ms for [1–^13^C]pyruvate, 30 ms for [1–^13^C]lactate, and 110 ms for ^13^C bicarbonate in the brain [14], and 60–120, 35–45, and 60–65 ms, respectively across kidney and heart [14, 45]. While relaxation differences in $T_{2}^{*}$ may influence absolute quantification, this phantom is designed to evaluate how image acquisition and analysis pipelines affect metabolite ratio measurements under controlled conditions.

### 4.2 Addressing a Gap

The need for a standardized ^13^C reference phantom, analogous to widely adopted ^1^H phantoms (ISMRM/NIST [21], ACR [19], ADNI [20]), has been repeatedly highlighted as HP ^13^C MRI transitions toward multicenter clinical studies [8].

The dynamic phantom designs emulating enzymatic conversion of [1–^13^C]pyruvate to [1–^13^C]lactate are physiologically appealing, yet inherently non‐standardizable due to chemical instability of [1–^13^C]pyruvate [26], variability in enzymatic activity, and repeated preparation requirements. For these reasons, dynamic phantoms are not suitable for routine QA or longitudinal multicenter calibration, where reproducibility and direct site comparison are the primary requirements. Static phantoms avoid these limitations but have traditionally been purpose‐specific, assessing specific aspects such as only field homogeneity, only geometry accuracy, or only spectral features [13]. Vendor provided natural‐abundance ^13^C phantoms are useful for field homogeneity and SNR assessment but cannot evaluate geometric fidelity, spatial resolution or pyruvate‐relevant spectral complexity. While ^1^H‐based QA procedures can partially assess gradient performance, they do not capture the full ^13^C‐specific imaging chain.

By consolidating these capabilities into a single standardized tool, this static ^13^C phantom directly addresses this gap, enabling harmonization and cross‐site comparison in ways not achievable with dynamic or single‐purpose static phantoms designs.

### 4.3 Limitations and Alternative Use Cases

To date, the phantom has been evaluated at a single site, using one scanner and one coil configuration. The current receive coil setup provides sufficient SNR primarily in regions closest to the receive elements, necessitating repositioning when imaging different regions. Multisite testing, including alternative coil configurations, will be essential to establish expected performance ranges and assess robustness. Several practical limitations were identified that inform future design iterations.

Relatively long scan times (26 min or more per module) may increase susceptibility to system drift, particularly on older scanners. Imaging protocol optimization and addition of ^13^C‐labeled urea in hemispheres could reduce acquisition time of the *uniform* hemisphere and the *geometric* and *resolution* modules. In the *quantification* module, the aim was to introduce different ratios in three different compartments to evaluate biases; however, at expense of SNR. Future versions may favor fewer, larger compartments with higher concentrations of the ^13^C‐labeled metabolites. Preliminary measurements indicate that glycine concentrations of approximately 3.2 M remain soluble at room temperature, including in the presence of sodium propionate and urea, supporting the feasibility of increasing metabolite concentrations by approximately 1.5‐fold while maintaining current ratios. Residual urea excitation outside the nominal imaging slice caused slice‐direction bleed‐through, affecting image quality and urea quantification in SPSP spiral imaging. Higher concentrations of ^13^C‐labeled urea in the *quantification* compartments or thicker modules may help mitigate this. Finally, air trapping occurred in narrow or angled compartments, potentially degrading image quality. Smoother geometries and modified filling procedures may should be investigated.

Beyond QA and harmonization, the phantom offers opportunities for methodological development in sequence design and verification, reconstruction benchmarking, or coil and hardware evaluation. In this work, ^13^C imaging tests were done using single‐slice 2D sequences, but the design of the phantom also allows for critical evaluation of volumetric imaging in the case of multislice or 3D sequences.

For sequence optimization, the phantom can be used to evaluate frequency‐dependent signal behavior and spectral selectivity. The presence of multiple compartments with well‐defined in vivo‐like frequency separations allows assessment of off‐resonance effects and frequency‐dependent signal modulation, providing a direct link between sequence design and image quality.

In reconstruction benchmarking, the phantom could potentially be used, for example, for assessment of compressed‐sensing strategies or multichannel combination methods under controlled, repeatable conditions. Finally, in coil design and comparison, the phantom could aid in standardized evaluation of novel coil geometries, particularly head coils given the phantom dimensions.

## 5 Conclusions

We developed a static phantom specifically designed to support harmonization of HP [1–^13^C]pyruvate MRI, showcasing features for assessing field and SNR homogeneity, geometric fidelity, actual spatial resolution, and metabolite ratio quantification. Future multisite testing will further validate its use across diverse scanner, coil and sequence configurations.

## Funding

This work was supported by ERC Synergy Grant HyperQ (856432), the Novo Nordisk Foundation (NNF 19OC0055825), LF Postdocs Grant (R449‐2023‐938), and EIC Transition Grant—RESPONSE (101158869).

## Conflicts of Interest

The authors declare no conflicts of interest.

## Supporting information


**Table S1.** ^13^C $T_{1}$, $T_{2}$, and $T_{2}^{*}$ measurements of natural abundance urea in the hemispheres at 3T and 11.7T.
**Figure S1.** Graphical comparison of $T_{1}$ measurements and fit for 11.7T (blue) and 3T (red) as described in Table S1.
**Table S2.** ^1^H $T_{1}$ and $T_{2}$ measurements of water in hemispheres at 3T.
**Table S3.** Initial measurements of $T_{1}$ and $T_{2}$ values (3T) for [1‐^13^C]glycine‐d_5_, [1‐^13^C]propionate, and ^13^C urea in 1‐mL containers to select Gd type (Omniscan, top or Dotarem, bottom) and appropriate amount of Gd doping, 7 or 22 mM.
**Table S4.** $T_{1}$ and $T_{2}$ values (3T) for [1‐^13^C]glycine‐d_5_, [1‐^13^C]propionate, and ^13^C urea mixed in 3.2‐mL container and doped with Dotarem (7 mM).
**Figure S2.** SNR map for each coil element (left) and combined (excluding ch1, right) using the developed ^13^C phantom.
**Figure S3.** SNR map for each coil element (left) and combined (excluding ch1, right) using vendor provided phantom (dimethyl silicone fluid phantom, manufactured for GE Healthcare by Dielectric Corp, USA).
**Figure S4.** $B_{0}$ map of vendor provided phantom (dimethyl silicone fluid phantom, manufactured for GE Healthcare by Dielectric Corp, USA).
**Figure S5.** $B_{1}^{+}$ map of vendor provided phantom (dimethyl silicone fluid phantom, manufactured for GE Healthcare by Dielectric Corp, USA).
**Figure S6.** Additional resolution module comparisons.

## Data Availability

The data that support the findings of this study are openly available in Zenodo at https://doi.org/10.5281/zenodo.18225650, reference number 18225650.
