# Supplementary material for: A Static Phantom for Harmonization of Hyperpolarized [1– 13C]Pyruvate Metabolic Magnetic Resonance Imaging
Source: Magn Reson Med. 2026 May 24;96(4):1769–77. doi: 10.1002/mrm.70442 (PMC13419006; doi:10.1002/mrm.70442)
Supplement: Supplementary file 1 — Table S1. 13C T1, T2, and T2* measurements of natural abundance urea in the hemispheres at 3T and 11.7T. Figure S1. Graphical comparison of T1 measurements and fit for 11.7T (blue) and 3T (red) as described in Table S1. Table S2. 1H T1 and T2 measurements of water in hemispheres at 3T. Table S3. Initial measurements of T1 and T2 values (3T) for [1‐13C]glycine‐d5, [1‐13C]propionate, and 13C urea in 1‐mL containers to select Gd type (Omniscan, top or Dotarem, bottom) and appropriate amount of Gd doping, 7 or 22 mM. Table S4. T1 and T2 values (3T) for [1‐13C]glycine‐d5, [1‐13C]propionate, and 13C urea mixed in 3.2‐mL container and doped with Dotarem (7 mM). Figure S2. SNR map for each coil element (left) and combined (excluding ch1, right) using the developed 13C phantom. Figure S3. SNR map for each coil element (left) and combined (excluding ch1, right) using vendor provided phantom (dimethyl silicone fluid phantom, manufactured for GE Healthcare by Dielectric Corp, USA). Figure S4. B0 map of vendor provided phantom (dimethyl silicone fluid phantom, manufactured for GE Healthcare by Dielectric Corp, USA). Figure S5. B1+ map of vendor provided phantom (dimethyl silicone fluid phantom, manufactured for GE Healthcare by Dielectric Corp, USA). Figure S6. Additional resolution module comparisons. [file MRM-96-1769-s001.docx]

Supporting Information

A Static Phantom for Harmonization of Hyperpolarized [1-^13^C]pyruvate Metabolic Magnetic Resonance Imaging

Kristina P. Jacobsen^1^, Ingeborg S. Skre^1^, Rie B. Olin^1^, Mathilde H. Lerche^1*^

^1^ Section for Magnetic Resonance, Department of Health Technology, Technical University of Denmark, Kgs. Lyngby, Denmark

^*^ Corresponding author: mhauler@dtu.dk, Oersteds Plads 349, Kgs. Lyngby

## Content:

- Stability tests of natural abundance urea in hemispheres
- Details of Imaging Tests for Characterization
- T_1_, T_2_, and T_2_* measurements of natural abundance urea in hemispheres
- T_1_ and T_2_ measurements of compounds in *quantification* module
- Coil characterization / SNR maps of developed phantom
- Coil characterization / SNR and field maps of vendor provided phantom
- Additional *resolution* module comparisons

## Stability tests of natural abundance urea in hemispheres

We investigated solubility and stability of natural abundance urea solutions for use in the phantom hemispheres, using both phosphate-buffered saline (PBS) and water as solvents.

Initial solutions were prepared across a concentration range of 6-9 M to assess solubility limits. Based on these preliminary tests, concentrations ≥7.5 M were selected for further evaluation. Density and solubility were examined, with no significant differences observed between PBS and water. PBS was selected as the solvent to ensure buffering capacity and compatibility with the intended application.

Stability testing was performed at both room temperature and at 4 °C. At 4 °C, precipitation was observed in 9 M solutions, whereas concentrations ≤8 M remained stable over the observation period. Based on these results, an 8 M urea solution in PBS was selected for use in the hemispheres as a compromise between maximizing signal and ensuring storage stability.

All stability assessments were conducted over repeated measurements spanning six months to confirm consistent solution behavior under the tested conditions.

Details of Imaging Tests for Characterization

**Positioning:**

The phantom was positioned with modules being in the axial plane, except for when the *quantification* module was imaged; here the module was placed in the coronal plane close to the Rx coil (most anterior). Foam padding and tape secured the Rx coil to the phantom.

**^1^H MR imaging:**

- B_0_ map: 2D GRE, FOV: 300 mm x 300 mm, slice thickness = 15 mm, TR = 250 ms, TE_min full_= 3.6 ms, FA = 35^◦^, receive bandwidth = 31.25 kHz, total scan time: 1 min, 6 seconds
- Structural images: 2D SPGR, FOV: 300 mm x 300 mm, slice thickness: 15 mm, TR = 11 ms, TE = 4.8 ms, FA = 53^◦^, matrix: 128 x 128, 10 averages, receive bandwidth = 31.25 kHz, total scan time: 14 seconds.

**^13^C MR imaging:**

- B_1_^+^ map: 2D CSI with off-resonance Bloch-Siegert pulses: FOV: 300 mm x 300 mm, slice thickness: 15 mm, matrix: 16 x 16, TR = 500 ms, TE = 6.279 ms FA = 90^◦^ (Ernst angle), pts = 1024, receive bandwidth = 5000 Hz, total scan time: 12 minutes, 48 seconds.
- SNR map: 2D CSI with similar imaging parameters as for B_1_^+^ mapping, except TE = 1.86 ms and FA = 70^◦^. (FOV: 300 mm x 300 mm, slice thickness: 15 mm, matrix: 16 x 16, TR = 500 ms, pts = 1024, receive bandwidth = 5000 Hz, total scan time: 25 minutes, 36 seconds)
- *Geometric* and *resolution* modules: 2D spectral-spatial (SPSP) spiral: spiral readout duration: 25.5 ms, FOV: 300 mm x 300 mm, slice thickness: 15 mm, matrix: 48 x 48, TR = 500 ms, TE = 9.865 ms, FA = 90^◦^, receive bandwidth = 125 kHz, total scan time: 25 minutes, 36 seconds. A spiral readout duration of 45.6 ms was tested on the *resolution* module for comparison.
- *Quantification* module:
  - SPSP spiral sequence: centered at the resonance frequencies of [1-^13^C]glycine-d_5_, [1-^13^C]propionate (+400 Hz), and ^13^C urea (-300 Hz). Modified parameters compared to other modules were TR = 5000 ms, TE = 9.161 ms, and total scan time: 56 minutes.
  - Phase-encoded CSI was acquired for comparison to SPSP spiral imaging: FOV: 100 mm x 100 mm, slice thickness: 15 mm, matrix: 20 x 20, TR = 5000 ms, TE = 1.871 ms, FA= 90^◦^, receive bandwidth = 5000 Hz, total scan time: 33 minutes, 20 seconds.
  - A single spectrum was acquired over the entire module: FOV: 300 mm x 300 mm, slice thickness: 15 mm, TR = 5000 ms, TE = 2.025 ms, FA = 90^◦^, receive bandwidth = 5000 Hz, total scan time: 10 minutes, 40 seconds.

**Reconstruction and analysis**

- Masking in B_1_^+^ maps was based on the standard deviation of the Bloch-Siegert phase with a threshold set to 60^◦^.
- *Resolution* module: Different postprocessing approaches: Gaussian line broadening of 0, 5, 10 Hz and GL-HOSVD with two different set of parameters:
  - GL-HOSVD: k_global_ = 0.4, k_local_ = 0.8, patch size = 5, step length = 2, search window size = 11.
  - Moderate GL-HOSVD: : k_global_ = 0.2, k_local_ = 0.3, patch size = 5, step length = 2, search window size = 10.
- *Quantification* module*:* AMARES fitting of CSI spectra: With bounds (prior knowledge): chemical shift: +/- 1 ppm of expected chemical shift value, linewidth: 0 – 20 Hz, amplitude: 0 – inf, phase: 0 – 360 degrees. Initial values were provided, and [1-^13^C]glycine-d_5_ sat as reference peak with no other prior knowledge provided for fitting.

## T_1_, T_2_, and T_2_* measurements of natural abundance urea in hemispheres

| **Urea** | **Measurement** | **Result** |
| --- | --- | --- |
| ^13^C T_1_ (ms)  (3 T) | Inversion recovery with TI = [20, 40, 60, 80, 100, 120, 140, 160, 200, 250, 300] ms.  Conducted on solutions before phantom filling: FOV = 200 x 200 mm^2^, slice thickness = 100 mm, TR = 1000 ms, TE = 0.455 ms, FA = 90^◦^, scan time: 32 seconds for each measurement. | T_1_ = 102 ± 1 ms (global value, n = 3) |
| ^13^C T_1_ (ms)  (11.7 T) | Confirmation of hemisphere urea T_1_ determined by inversion recovery with TI = [4, 8, 16, 32, 62.5, 125, 250, 500, 1000, 2000] ms, and TR = 5000 ms and 10 averages per measurement.  Conducted on solution NMR sample before phantom filling. | T_1_ = 134 ± 5 ms (n = 3) |
| ^13^C T_2_ (ms)  (3 T) | Spin echo with TE = [32, 40, 50, 60, 80, 100, 120, 150, 200, 250] ms.  Conducted on solutions before phantom filling: FOV = 120 x 120 mm^2^, slice thickness = 80 mm, TR = 1000 ms, FA = 90^◦^, scan time: 40 seconds for each measurement. | T_2_ = 53 ± 2 ms (global value, n = 3) |
| ^13^C T_2_* (ms)  (3 T) | Slice-selective imaging on *Uniform hemisphere* and *Resolution* module with TE = [1.871, +10, +20, +30, +40, +80, +120] ms. Other scan parameters: FOV: 300 mm x 300 mm, slice thickness: 15 mm, matrix: 48 x 48, TR = 500 ms, FA = 90^◦^, total scan time 32min:24sec (*uniform* hemisphere) and 64min:50sec for *resolution* module. ROI selection was based on SNR above 4. | T_2_^*^ = 50 ± 7 ms (n = 2)  (standard deviation: ~15 ms across *Uniform* hemisphere slice for both measurements)  T_2_^*^ = 28 ms (n = 1) (standard deviation: ~5 ms across *Resolution* module) |

**Table S1**: ^13^C T_1_, T_2_, and T_2_* measurements of natural abundance urea in the hemispheres at 3 T and 11.7 T. *n* represents number of measurements.


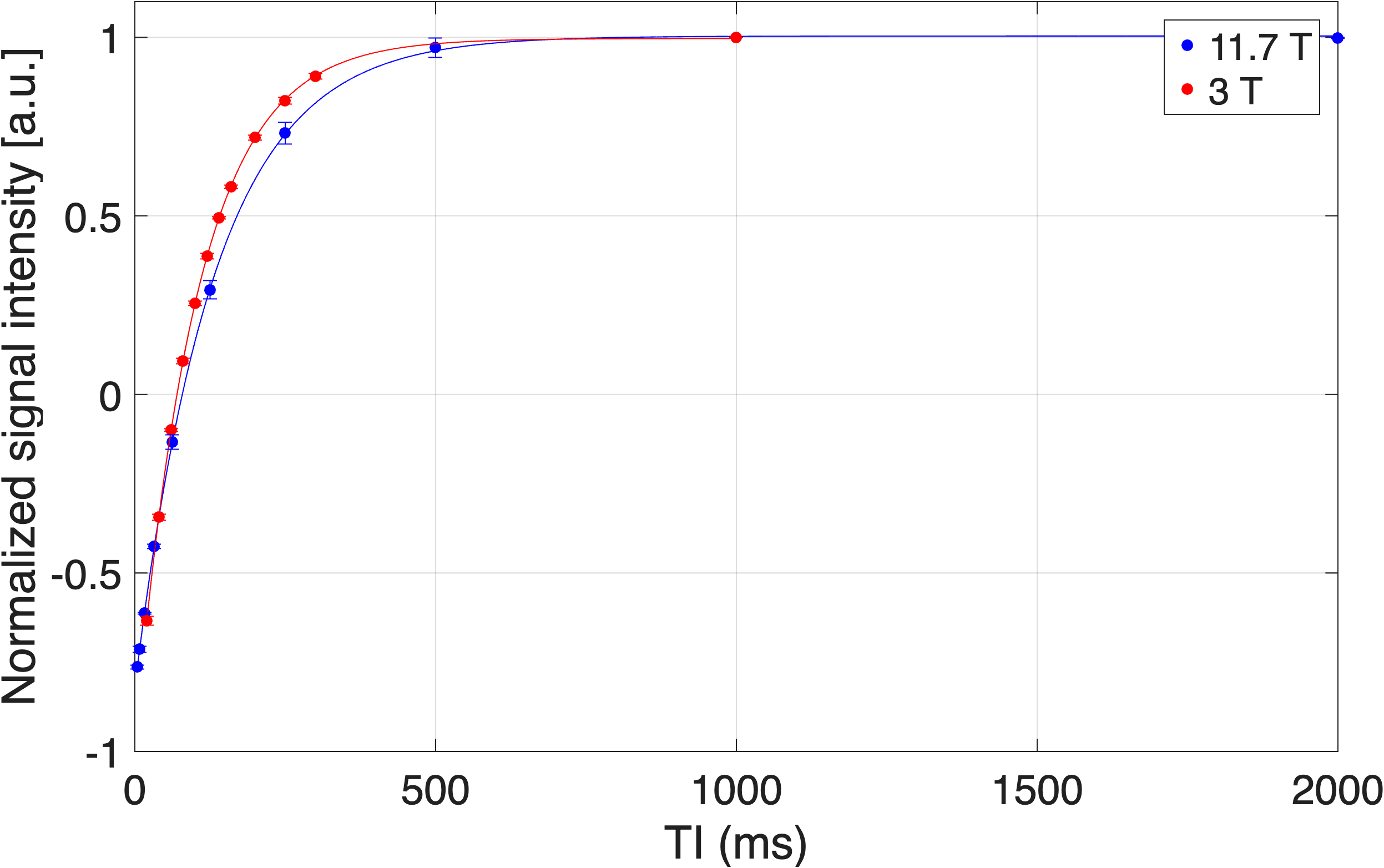


**Figure S1:** Graphical comparison of T1 measurements and fit for 11.7 T (blue) and 3 T (red) as described in Table S1 (n = 3 for both measurements).

| **Water** | **Measurement** | **Result** |
| --- | --- | --- |
| ^1^H T_1_ (ms) (3 T) | Inversion recovery with TI = [20, 30, 40, 50, 60, 70, 100, 300] ms.  Conducted on the entire phantom: FOV = 300 x 300 mm^2^, slice thickness = 300 mm, TR = 2000 ms, TE = 0.455 ms, FA = 90^◦^, scan time: 16 seconds for each measurement. | T_1_ ~20 ms (global value) (n = 2) |
| ^1^H T_2_ (ms) (3 T) | Vendor provided T2 Map sequence. 2D FSE: TR = 1000 ms, FA = 90^◦^, slice thichness = 15 mm, FOV = 200 x 200 mm^2^, matrix = 256 x 256. TE = [7.808, 15.616, 23.424, 31.232, 39.040, 46.848, 54.656, 62.464] ms. | T_2_ ~ 14 ms (mean value across slice) (n = 3) |

**Table S2:** ^1^H T_1_ and T_2_ measurements of water in hemispheres at 3 T. *n* represents number of measurements.

## T_1_ and T_2_ measurements of compounds in *quantification* module

To determine amount of Gd doping, T_1_ and T_2_ measurements were done at 3 T on separate containers with a single compound (1 mL) – Table S3. Both Omniscan and Dotarem were tested. The design criteria were T_1_ < 1000 ms to allow practical scan durations, and T_2_ values > 50 ms to preserve signal, with T_2_ also varying between compounds to allow differential assessment.

- For T_1_ estimation, inversion recovery was done with TI values ranging from 20 ms to 4000 ms depending on compound.
- For T_2_ estimation, TE values in spin echo measurements ranged from 32 ms to 250 ms.

| **Omniscan** | **[1-^13^C]glycine-d_5_** | **[1-^13^C]propionate** | **^13^C urea** |
| --- | --- | --- | --- |
| **T₁ (ms):** |  |  |  |
| 7 mmol/L | 121 | 375 | 130 |
| 22 mmol/L | N/A | 113 | 43 |
| **T₂ (ms):** |  |  |  |
| 7 mmol/L | 50 | 68 | 55 |
| 22 mmol/L | N/A | 45 | 26 |

| **Dotarem** | **[1-^13^C]glycine-d_5_** | **[1-^13^C]propionate** | **^13^C urea** |
| --- | --- | --- | --- |
| **T₁ (ms):** |  |  |  |
| 7 mmol/L | 394 | 925 | 142 |
| 22 mmol/L | N/A | N/A | N/A |
| **T₂ (ms):** |  |  |  |
| 7 mmol/L | 122 | 84 | 51 |
| 22 mmol/L | N/A | N/A | N/A |

**Table S3:** Initial measurements of T_1_ and T_2_ values (3 T) for [1-^13^C]glycine-d_5,_ [1-^13^C]propionate, and ^13^C urea in 1 mL containers to select Gd type (Omniscan, top or Dotarem, bottom) and appropriate amount of Gd doping, 7 mM or 22 mM. Number of measurements: n = 1 for all measurements.

Dotarem was selected over Omniscan as initial T₂ measurements with Omniscan showed limited differentiation between the compounds. To validate the relaxation values with Dotarem (7 mM) under conditions matching the *quantification* module, measurements were repeated after volume scaling and mixing the compounds to a total of 3.2 mL, as summarized in Table S4.

| Dotarem | **[1-^13^C]glycine-d_5_:** | **[1-^13^C]propionate:** | **^13^C urea:** |
| --- | --- | --- | --- |
| T1 (ms)  - 7 mmol/L in 3.2 mL | 304 ± 14 (n=3) | 799 ± 36 (n=3) | 226 ± 6 (n=3) |
| T2 (ms)  - 7 mmol/L in 3.2 mL | 117 ± 5 (n=3) | 73 ± 1 (n=3) | 55 ± 3 (n=3) |

**Table S4**: T_1_ and T_2_ values (3 T) for [1-^13^C]glycine-d_5,_ [1-^13^C]propionate, and ^13^C urea mixed in 3.2 mL container and doped with Dotarem (7 mM). Relaxation values are listed as mean ± standard deviation, and *n* is denoting number of measurements.

## Coil characterization / SNR maps of developed phantom


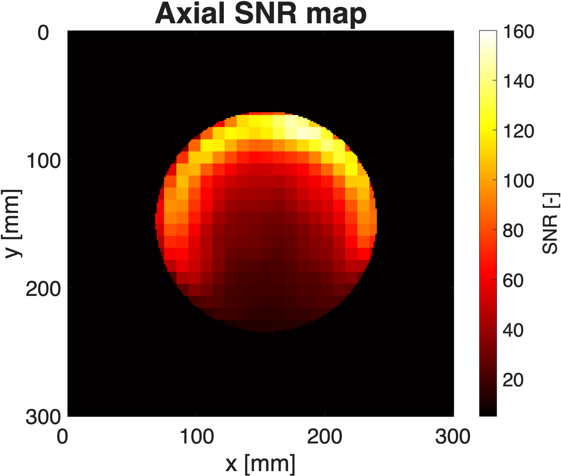


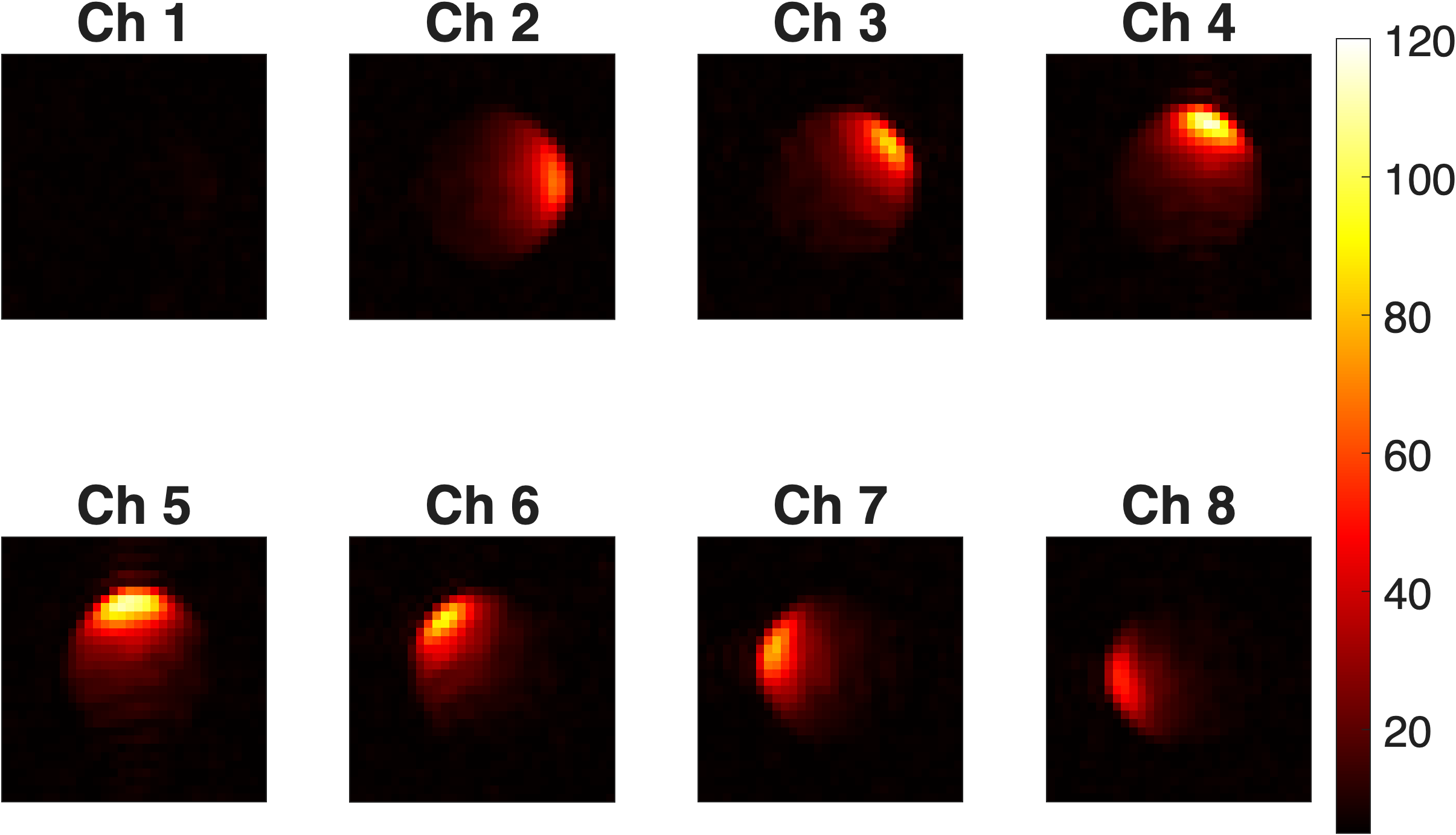


**Figure S2.** SNR map for each coil element (left) and combined (excluding ch1, right) using the developed ^13^C phantom. FOV: 300 mm x 300 mm, slice thickness: 15 mm, TR = 500 ms, TE = 1.86 ms, FA = 70^◦^, bw = 5000 Hz, pts = 1024, NEX = 12, total scan time: 25 minutes, 36 seconds. Ch: coil channel; SNR: signal-to-noise ratio.

## Coil characterization / SNR and field maps of vendor provided phantom


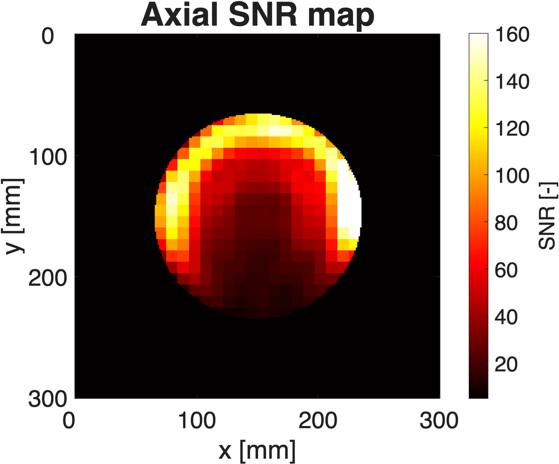


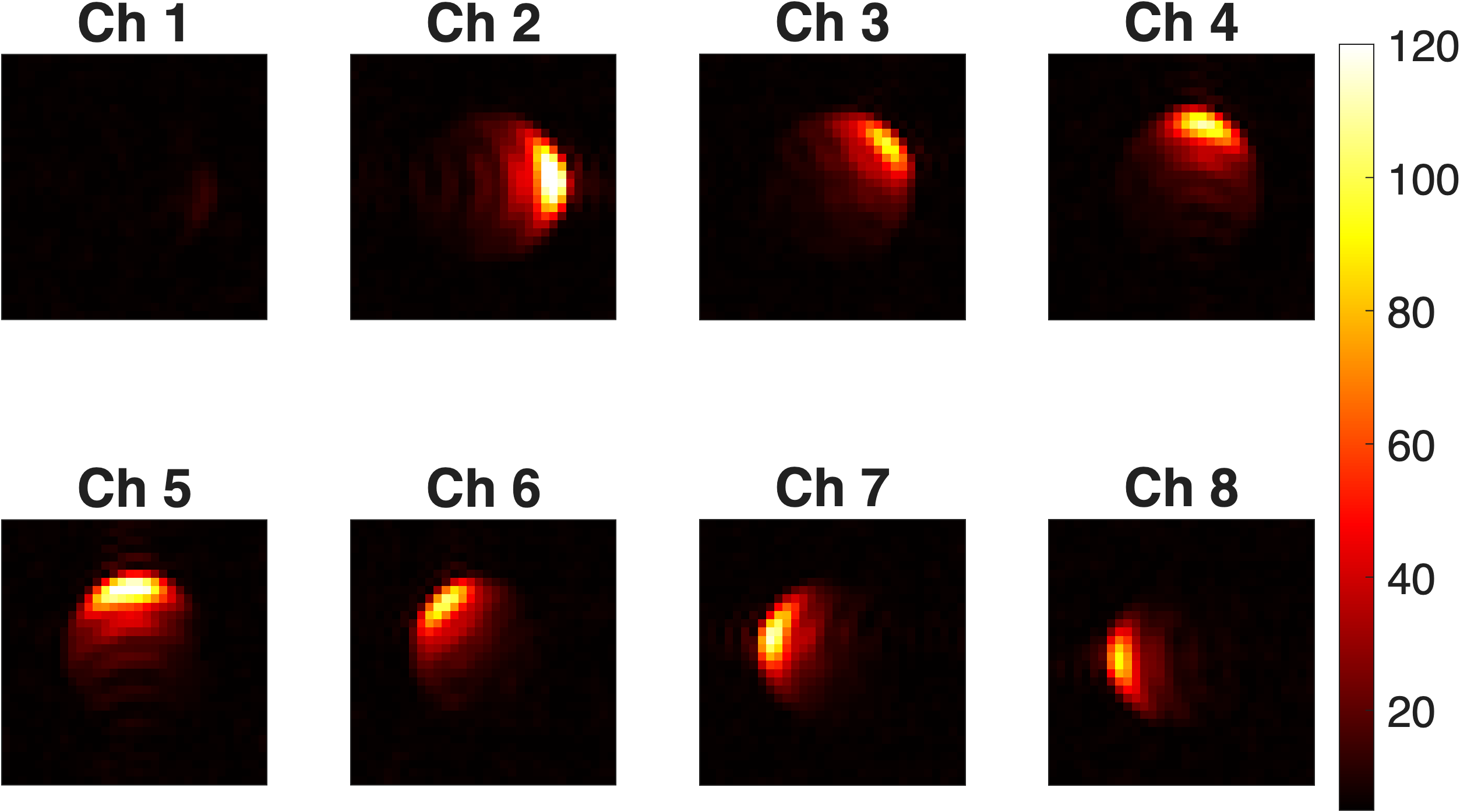


**Figure S3.** SNR map for each coil element (left) and combined (excluding ch1, right) using vendor provided phantom (dimethyl silicone fluid phantom, manufactured for GE Healthcare by Dielectric Corp, USA). FOV: 300 mm x 300 mm, slice thickness: 15 mm, TR = 500 ms, matrix: 16 x16, TE = 1.86 ms, FA = 30^◦^, bw = 5000 Hz, pts = 1024, NEX = 12, total scan time: 25 minutes, 36 seconds. Ch: coil channel; SNR: signal-to-noise ratio.


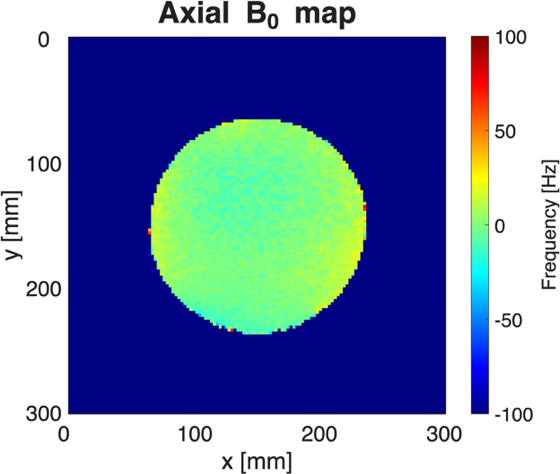


**Figure S4.** B_0_ map of vendor provided phantom (dimethyl silicone fluid phantom, manufactured for GE Healthcare by Dielectric Corp, USA). 2D GRE, FOV: 300 mm x 300 mm, slice thickness = 15 mm, TR = 250 ms, TE_min full_ = 3.6 ms, FA = 35^◦^, total scan time: 1 min, 6 sec.


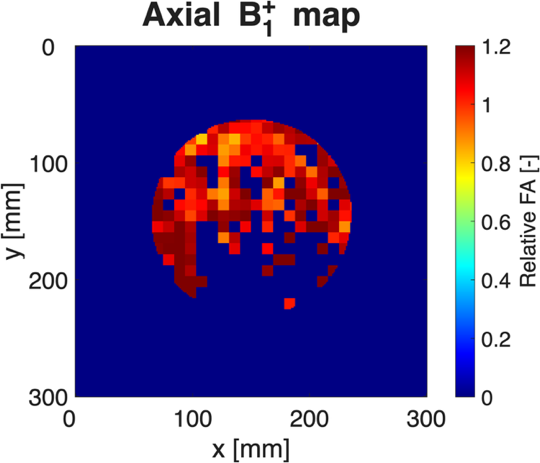


**Figure S5** B_1_^+^ map of vendor provided phantom (dimethyl silicone fluid phantom, manufactured for GE Healthcare by Dielectric Corp, USA). FOV: 300 mm x 300 mm, slice thickness: 30 mm, TR = 500 ms, matrix: 16 x 16, TE = 5.792 ms, FA = 50^◦^ (Ernst angle), bw = 5000 Hz, pts = 1024, NEX = 12, total scan time: 51 minutes, 12 seconds.

# Additional *resolution* module comparisons

**
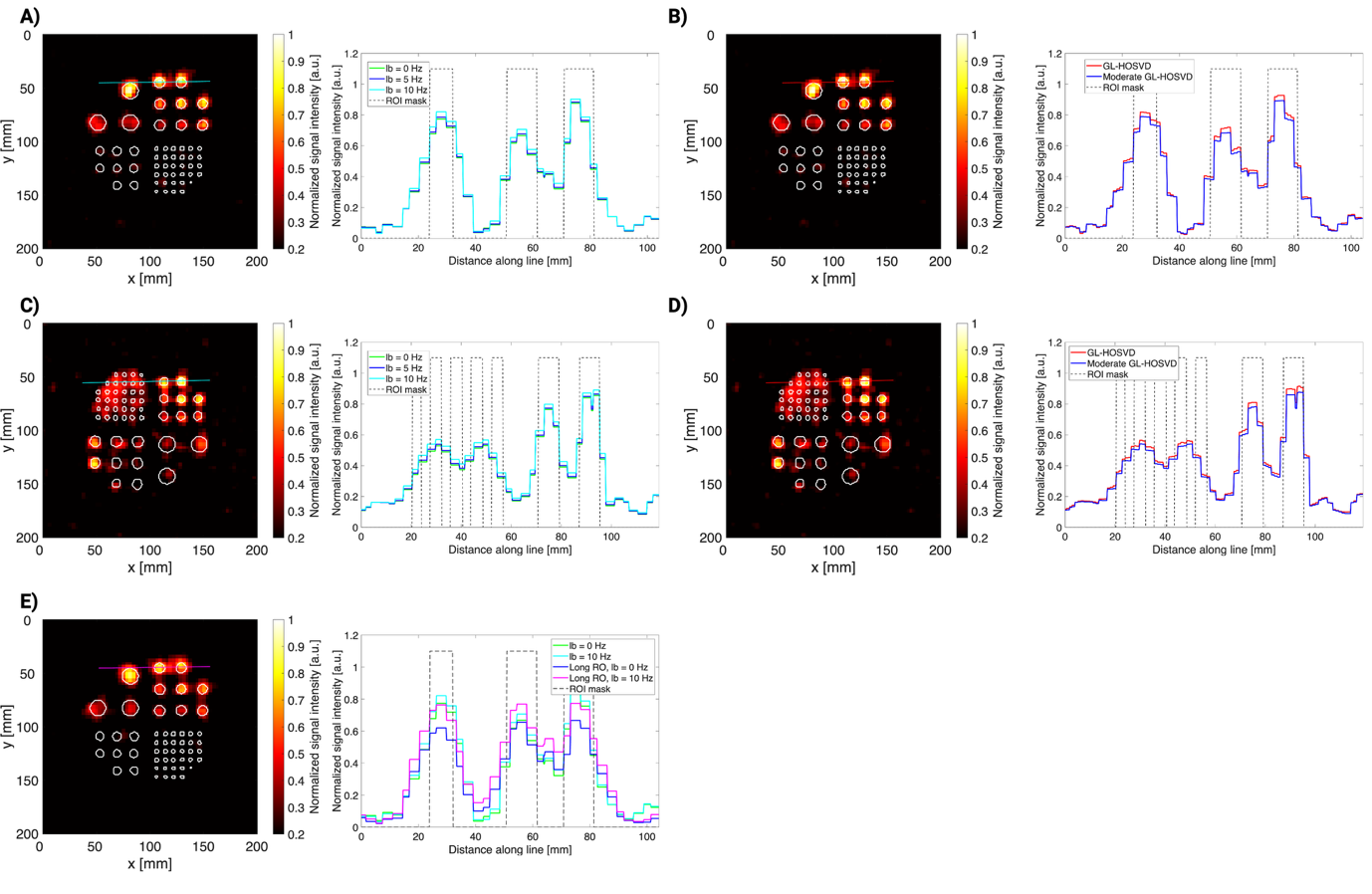
Figure S6.** Additional *resolution* module comparisons. A, C) ^13^C images with 10 Hz line broadening and corresponding ^1^H reference (white), indicating large (Ø15 mm, Ø10 mm) and small (Ø4 mm, Ø8 mm) holes. Blue line profiles through the holes are shown beside the images and compared with profiles from 0 Hz and 5 Hz line broadening. Black dashed lines indicate hole locations derived from the ^1^H reference. B, D) ^13^C images with GL-HOSVD denoising and ^1^H references (large and small holes as above). Red line profiles through the holes are shown beside the images compared to other “moderate” GL-HOSVD denoising parameters, with black dashed lines indicating hole locations. E) ^13^C image from spiral acquisition with longer read-out duration (45.6 ms) with 10 Hz line broadening with line profile comparison to the standard acquisition (25.5 ms).
